# Direct Growth of Wafer‐Scale Self‐Separated GaN on Reusable 2D Material Substrates

**DOI:** 10.1002/advs.202406126

**Published:** 2024-09-03

**Authors:** Chang‐Hsun Huang, Chia‐Yi Wu, Yi‐Chia Chou

**Affiliations:** ^1^ Department of Material Science and Engineering National Taiwan University Taipei 10617 Taiwan

**Keywords:** gallium nitride, hydride vapor‐phase epitaxy, self‐separation, 2D materials, van der Waals epitaxy

## Abstract

Free‐standing gallium nitride has been prepared using various methods; however, the removal of the original substrate is still challenging with low success rates. In this work, 2‐inch free‐standing GaN films are obtained by direct growth on a fluoro phlogopite mica by hydride vapor‐phase epitaxy. Depending on the van der Waals (vdW) interaction between GaN and mica, the effect of the significant lattice mismatch is effectively reduced; thus, enabling the production of a high‐quality wafer‐scale GaN film on mica. The vdW‐induced cracks at GaN–mica interface are found to be initiated near the interface so that GaN can easily separate from mica during rapid cooling. Owing to the hydrophilic nature of mica, the residual GaN on the mica can be lifted off by following deionized water treatment, and the mica substrate can be repeatedly used to grow free‐standing GaN films. The self‐separated GaN films grown on both pristine and used mica substrates are single crystallinity and strain‐free. Additionally, a fully functional ultraviolet light‐emitting diode is demonstrated to show that the self‐separated GaN films are of device quality. The proposed approach achieves epitaxial growth of wafer‐scale single‐crystalline GaN on 2D materials and provides a new substrate option in the technology of III‐V materials.

## Graphical abstract

A self‐separation technique for generating thick GaN films on a 2D material substrate is demonstrated for the first time. The reusability of mica substrates by repeatedly growing thick GaN films on the same substrate is shown. Moreover, ultraviolet light‐emitting diodes on self‐separated thick GaN films, demonstrating the device quality of these thick GaN films are fabricated.

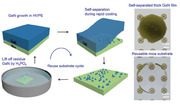

## 1 Introduction

Gallium nitride (GaN) is widely used in light‐emitting diodes (LEDs),^[^ ^1^, ^2^ ^]^ high‐power electronic devices,^[^ ^3^, ^4^ ^]^ and lasers^[^ ^5^, ^6^ ^]^ owing to its wide direct band gap and good chemical stability. In general, GaN‐based devices are heteroepitaxial grownon silicon,^[^ ^7^, ^8^ ^]^ sapphire,^[^ ^9^, ^10^ ^]^ or silicon carbide^[^ ^11^, ^12^ ^]^ substrates because of the difficulty in preparing high‐quality GaN substrates. The heteroepitaxial growth of GaN films on such substrates is associated with a high defect density and high biaxial strain, attributed to the significant lattice and thermal mismatch between GaN films and their substrates.^[^ ^13^ ^]^ Compared with the metal‐organic chemical vapor deposition (MOCVD) technique, the hydride vapor‐phase epitaxy (HVPE) method, which has high growth rates (100 µm h^−1^ for GaN epitaxy), can be used to deposit high‐quality thick GaN films with relatively low cost.^[^ ^14^ ^]^ Although several methods exist for obtaining free‐standing GaN substrates, such as the laser lift‐off process,^[^ ^15^ ^]^ void‐assisted separation,^[^ ^16^ ^]^ tape‐assisted separation,^[^ ^17^, ^18^ ^]^ and chemical etching of the intermediate layer between GaN and the original substrate,^[^ ^19^ ^]^ the process of removing the original substrate from the thick GaN film is challenging and is associated with drawbacks such as high complexity, lengthy process, and high cost.

Recently, the van der Waals epitaxial (vdWE) growth of III‐V films on 2D materials has been demonstrated to effectively diminish the lattice mismatch effect owing to the weak bonding between III‐V and 2D materials.^[^ ^20^, ^21^ ^]^ Utilizing the benefits of weak vdWE bonding, it is possible to generate free‐standing single‐crystalline films using 2D material‐assisted transfer techniques, which is termed “remote epitaxy.”^[^ ^22^, ^23^ ^]^ The epitaxially grown films are easily peeled away from the substrate owing to the weak vdW bonding of 2D materials, leaving a clean surface after exfoliation. However, the area yield of exfoliation is reliant on the quality and flatness of the transferred 2D material layer.^[^ ^24^ ^]^ In terms of emerging 2D material substrates, fluoro phlogopite mica (hereinafter referred to as “mica”) is a thermally stable material with a flexible atomically flat surface, wide commercial availability, and high‐quality wafer‐scale level, making it an ideal 2D material substrate.^[^ ^25^ ^]^ However, the major obstacle is that the dangling bond‐free surface of 2D materials enhances the barrier of III‐V nucleation, hence restricting the growth of large‐area III‐N with a single crystallinity.^[^ ^26^ ^]^ Although graphene has been recently proposed as an intermediate layer in bridging techniques for the large‐area growth and lift‐off of III‐V films,^[^ ^27^, ^28^ ^]^ the integration of directly grown wafer‐scale GaN and mica substrate from which GaN can be self‐separated remains relatively unexplored.

In this study, we successfully grew two‐inch thick GaN films using HVPE on a fluorophlogopite mica substrate with a high growth rate of up to 97 µm h and realized the self‐separation of the thick GaN film from the mica substrate. The influence of temperature on the mica substrate subjected to annealing was investigated. During the rapid cooling process, the as‐grown thick GaN film easily self‐separated from the mica substrate. The GaN–mica interface was further investigated to elucidate the self‐separation mechanism. After the growth of the self‐separated GaN, the residual GaN on the separated mica substrate could be efficiently removed by immersing the substrate in deionized (D.I.) water owing to the hydrophilic nature of mica and the weak bonding between residual GaN and mica. This process avoids the complexity and damage associated with lift‐off procedures, providing benefits for the III‐V semiconductor industry. Furthermore, we demonstrated the reusability of the mica substrates by growing thick GaN films repeatedly on the used mica substrate. To prove that the self‐separated thick GaN film was of device quality, we demonstrated a fully functional ultraviolet (UV) LED. Overall, our proposed approach offers valuable insights into the epitaxial growth of large‐area single‐crystalline GaN on 2D materials and provides a new substrate option in the self‐separation technology of III‐V materials.

## 2 Results and Discussion

### 2.1 Growth Procedure of Self‐Separated Thick GaN Film on Mica Substrate

**Figure 1:**
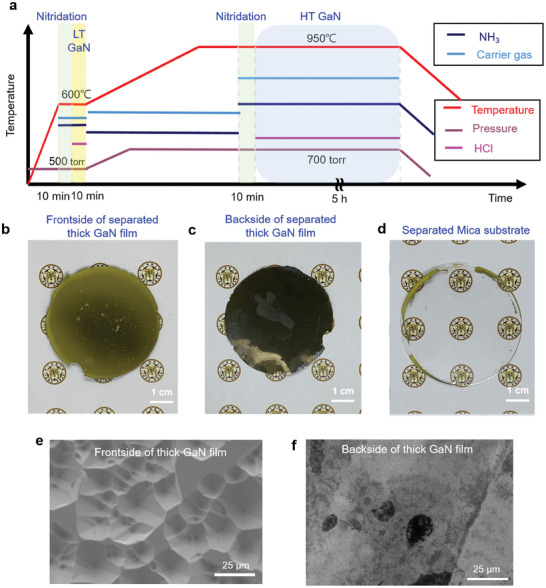
Fabrication process and images of 2‐inch self‐separated thick GaN film. a) Schematic of the fabrication process of a self‐separated thick GaN film. b) A 2‐inch self‐separated thick GaN film with an average thickness of 483 µm. c) Back side of the 2‐inch self‐separated thick GaN film. d) Mica substrate separated from the GaN film with a thickness of 28 µm. e, f) Plane‐view SEM images of the front and back sides of the self‐separated thick GaN film, respectively.

To study the influence of temperature and precursors on fluorophlogopite mica, we examined each step of the GaN growth process in the HVPE system. **Figure** **1**a shows a schematic of the procedure of the growth duration sequence and growth temperature of the self‐separated thick GaN film grown using the HVPE method. Figure S1a, Supporting Information, shows the 2‐inch pristine fluorophlogopite mica, which can be exfoliated to obtain an average thickness of 32 ± 5 µm, and Figure S1b, Supporting Information, represents the X‐ray photoelectron spectroscopy (XPS) spectrum of pristine mica surface. Prior to GaN growth, evaluating the surface morphology of the mica substrate after nitridation is crucial. Figure S2a,b, Supporting Information, show the morphologies of the nitridated mica surface at temperatures of 600 °C and 950 °C, respectively; Figure S2c,d, Supporting Information, show the corresponding XPS spectra, respectively. No pits were observed in the sample nitridated at 600 °C, and the elements in the mica substrate remained without any shift in the binding energy. However, several pits could be found on the mica surface nitridated at 950 °C. Ammonia attacks the defects on the mica surface, leading to several surface pits at high temperatures, which have also been noted in graphene.^[^ ^29^, ^30^ ^]^ With the F atoms being relatively light and intrinsically volatile, the absence of F indicates that the F atoms were dissipated during nitridation at 950 °C, possibly because the outermost F atoms are seized on the surface by weak ionic bonding with the anionic aluminosilicate layers, which can be easily broken by high‐temperature annealing.^[^ ^31^ ^]^ Further, the mica substrate deteriorates easily on annealing at temperatures above 1000 °C, as shown in Figure S3, Supporting Information, leading to the deterioration of the GaN grown on the mica substrate.

Taking this into account, we introduced a 2‐step low temperature (LT)/high temperature (HT) GaN growth process on mica to prevent serious damage to the substrate. As shown in the scanning electron microscopy (SEM) images of LT GaN in Figure S4a, Supporting Information, LT GaN can be uniformly grown on the mica substrate without separation. The cross‐sectional view of the SEM image shows that the thickness of LT GaN is 347 nm with high uniformity, as shown in Figure S4b, Supporting Information. After being annealed at 950 °C for 30 min, the LT GaN shows a morphology characterized by coalescence islands but still maintains a similar thickness (Figure S4c,d, Supporting Information). After 5 h of HT GaN growth after LT GaN, a two‐inch thick GaN film with an average thickness of 483 ± 25 µm was grown. With rapid cooling after HT GaN growth, the thick GaN film self‐separated from the mica substrate. Figure 1b shows the two‐inch thick GaN film self‐separated from the mica substrate, where the mica substrate after GaN growth is referred to as the “used” mica substrate. The back side of the self‐separated thick GaN film is heavily covered in adhesive mica flakes derived from the mica substrate, and the darker color of the back side of the self‐separated thick GaN film reflects the low quality of LT GaN (Figure 1c). The used mica substrate exhibits residual GaN islands originating from the thick GaN film on the mica surface, which is shown in Figure 1d. The thicknesses of the self‐separated thick GaN film and used mica substrate were also measured for wafer‐scale uniformity (Figure S5, Supporting Information). To further observe the morphology, we captured the SEM images of both the front and back sides of the self‐separated thick GaN film, as shown in Figure 1e,f, respectively. A rougher surface than that seen on the GaN films grown using the MOCVD method can be observed on the front side of the separated GaN (Figure 1e); this is attributed to the etching effect of the HCl precursor during the HT growth of GaN in the HVPE system.^[^ ^32^ ^]^ By contrast, a smoother surface with a whiter contrast is observed on the back of the separated thick GaN film (Figure 1f). Notably, the energy‐dispersive X‐ray spectroscopy (EDS) spectra of the back side of the thick GaN film help confirm that it is residual adhesive mica (Figure S6, Supporting Information). The element mapping of EDS also proves that the composition of the residual adhesive mica remains unchanged. Furthermore, the polarity of the polished self‐separated thick GaN film etched in phosphoric acid at 150 °C for 5 min was investigated. The morphologies of the front and back surfaces (Figure S7, Supporting Information) after etching N‐polar GaN can be identified when etching in phosphoric acid (H_3_PO_4_) at 150 °C at a higher etching rate; this is because the etching rate and the resulting surface morphology are polarity dependent.^[^ ^33^ ^]^ As shown in Figure S7, Supporting Information, the front of the self‐separated thick GaN film is Ga‐polar GaN. Previous studies have shown that N atoms prefer to distribute on the surface of 2D materials due to the lower nucleation of III‐V,^[^ ^34^, ^35^ ^]^ which is consistent with our result. Additionally, we fabricate GaN on mica substrate using the same growth recipe but omitting the rapid cooling process, resulting in the GaN film adhering to the mica substrate even when bent (Figure S8, Supporting Information). This observation underscores the vital role of the rapid cooling process in enabling the GaN film to self‐separate from the mica substrate.

### 2.2 Characterization of 2‐Inch Self‐Separated Thick GaN Film

**Figure 2:**
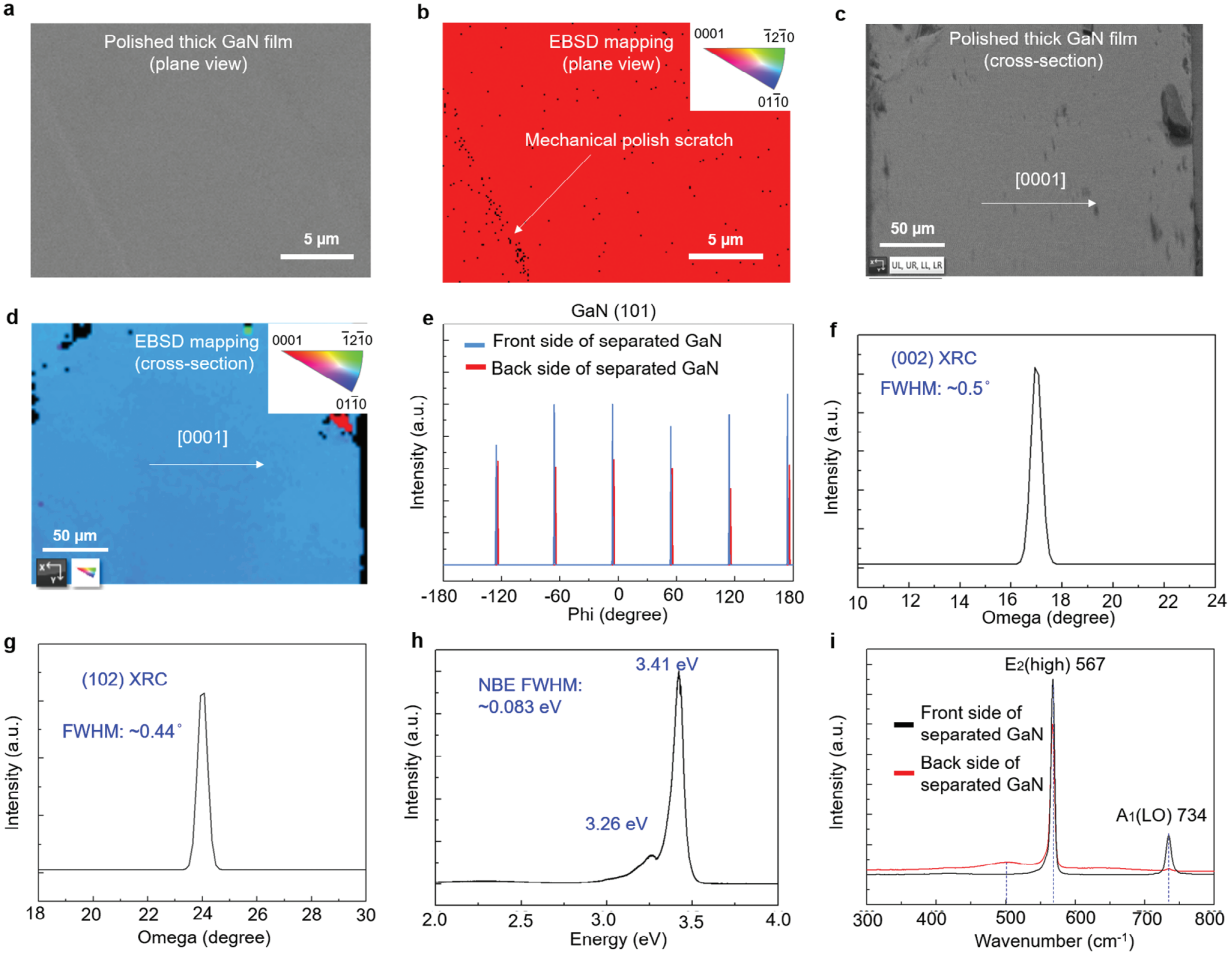
Morphology and crystalline quality of thick GaN film self‐separated from a mica substrate. a) Plane‐view SEM image of a polished self‐separated thick GaN film. b) EBSD mapping of (a). The inset shows the corresponding crystal orientation from the EBSD mapping. c) Cross‐sectional SEM image of the polished self‐separated thick GaN film. d) EBSD mapping of (c). The inset shows the corresponding crystal orientation from the EBSD mapping. e) XRD phi‐scan of the polished self‐separated GaN showing a single in‐plane alignment. f,g) (002) and (102) XRC of separated thick GaN film, with FWHM values of 0.5° and 0.44°, respectively. h) 10 K PL spectrum of the separated thick GaN film, and the FWHM of the near‐band edge (NBE) emission peak is 0.083 eV. i) Raman spectra of the front side (black line) and backside (red line) of the separated thick GaN film.

We examined the crystal quality and strain release of the self‐separated thick GaN film. **Figure** **2**a shows that the self‐separated thick GaN film can be subjected to chemical and mechanical polishing until it becomes a flat surface for further application in III‐V electronic devices. The crystallinity was confirmed through electron backscatter diffraction (EBSD) mapping, indicating that the separated thick GaN film is a single crystal with a c‐orientation (Figure 2b). Some of the black dots seen in Figure 2b can be attributed to the lattice damage induced during mechanical polishing. Figure 2c shows the cross‐sectional SEM image of the polished self‐separated GaN, which was used for the EBSD mapping measurement. The EBSD mapping shows that the cross‐sectional orientation observed is (11‐20) planar with single crystallinity (Figure 2d). The single crystallinity of GaN can also be confirmed from the XRD pattern of the polished self‐separated GaN, which exhibits six‐fold symmetric GaN lattices devoid of polycrystalline phases (Figure 2e). Figure 2f,g show the (002) and (102) X‐ray rocking curves (XRC) of the separated GaN film, respectively. The XRC of the self‐separated GaN film with a thickness of 483 µm exhibits a full width at half maximum (FWHM) of 0.5°, with the value being 1800 arcsec for the separated thick GaN film; however, the FWHM of the (102) XRC decreases to 0.44°, with the value being 1584 arcsec for the separated thick GaN film. The densities of screw dislocations and edge dislocations reportedly correspond to the FWHM values of the (002) and (102) XRCs, respectively.^[^ ^36^ ^]^ From the XRC FWHM values, the densities of screw‐type dislocation *D_s_* and the edge‐type dislocation *D_e_* can be calculated using the following formulae:^[^ ^37^ ^]^

(1) $$D_{s}=\frac{{\beta_{s}}^{2}}{4.35\times {\left|b_{s}\right|}^{2}}=\frac{{\beta_{002}}^{2}}{4.35\times {\left(b_{s}\cos\alpha \right)}^{2}}$$

(2) $$D_{e}=\frac{{\beta_{e}}^{2}}{4.35\times {\left|b_{e}\right|}^{2}}=\frac{{\beta_{102}}^{2}-{\beta_{002}}^{2}}{4.35\times {\left(b_{e}\cos\alpha \right)}^{2}}$$

Here, |b_s_| and |b_e_| are the Burgers vector magnitudes of the screw and edge dislocations, respectively. *β*_002_ and *β*_102_ are the XRD FWHM values for the (002) and (102) planes, respectively. α represents the angle between the reciprocal lattice vector and the normal of the (001) surface. For the self‐separated thick GaN film, the calculated screw dislocation density is 1.4 × 10^9^ cm^−2^, whereas the edge dislocation density is only 5.1 × 10^8^ cm^−2^. Notably, the edge‐type dislocations in GaN can be effectively reduced through the buffer layers in a 2D material.^[^ ^38^ ^]^ GaN nuclei only prefer to locate at defect sites on the mica surface, and the Ga and N adatoms easily migrate on the dangling bond‐free surface of mica, leading to lateral growth of GaN on the mica surface. Therefore, the lower density of GaN islands and higher affinity for lateral growth contribute to a lower edge dislocation density. Figure 2h shows the 10 K PL spectrum of the front side of the self‐separated thick GaN film. Although the threading dislocation values calculated were higher than those reported for self‐separated GaN, a strong near band‐edge emission (NBE) peak of GaN could be detected at 3.41 eV, and the FWHM of the NBE peak was 83 meV. Another PL peak occurred at 3.26 eV, attributed to oxygen impurities^[^ ^39^ ^]^ originating from the low‐vacuum environment in the HVPE chamber and the mica substrate.

Raman spectroscopy offers information on the vibrational states of GaN that are sensitive to stress. The position of the E_2_ phonon in a strain‐free GaN bulk is believed to be 567 cm^−1^. We calculated the compressive stress under relaxation based on this value using the equation:^[^ ^40^ ^]^

(3) $$\sigma =\frac{\Delta \omega }{4.2}\left(cm^{-1}GPa^{-1}\right)$$

Here, *σ* is the biaxial stress, and Δω is the E_2_ phonon peak shift. In Figure 2i, the *E*_2_ phonon peaks of the front and back sides of the self‐separated thick GaN film are both located at 567 cm^−1^, meaning that no residual strain exists on either side of the self‐separated thick GaN film. The A_1_(LO) peak also remains at 734 cm^−1^, as shown in Figure 2i, providing strong evidence of residual strain release. Furthermore, a slight peak appears at 500 cm^−1^ in Figure 2i, which is attributed to the residual mica that adhered to the back side of the separated GaN.

### 2.3 Interface Between Self‐Separated Thick GaN Film and Residual Mica

**Figure 3:**
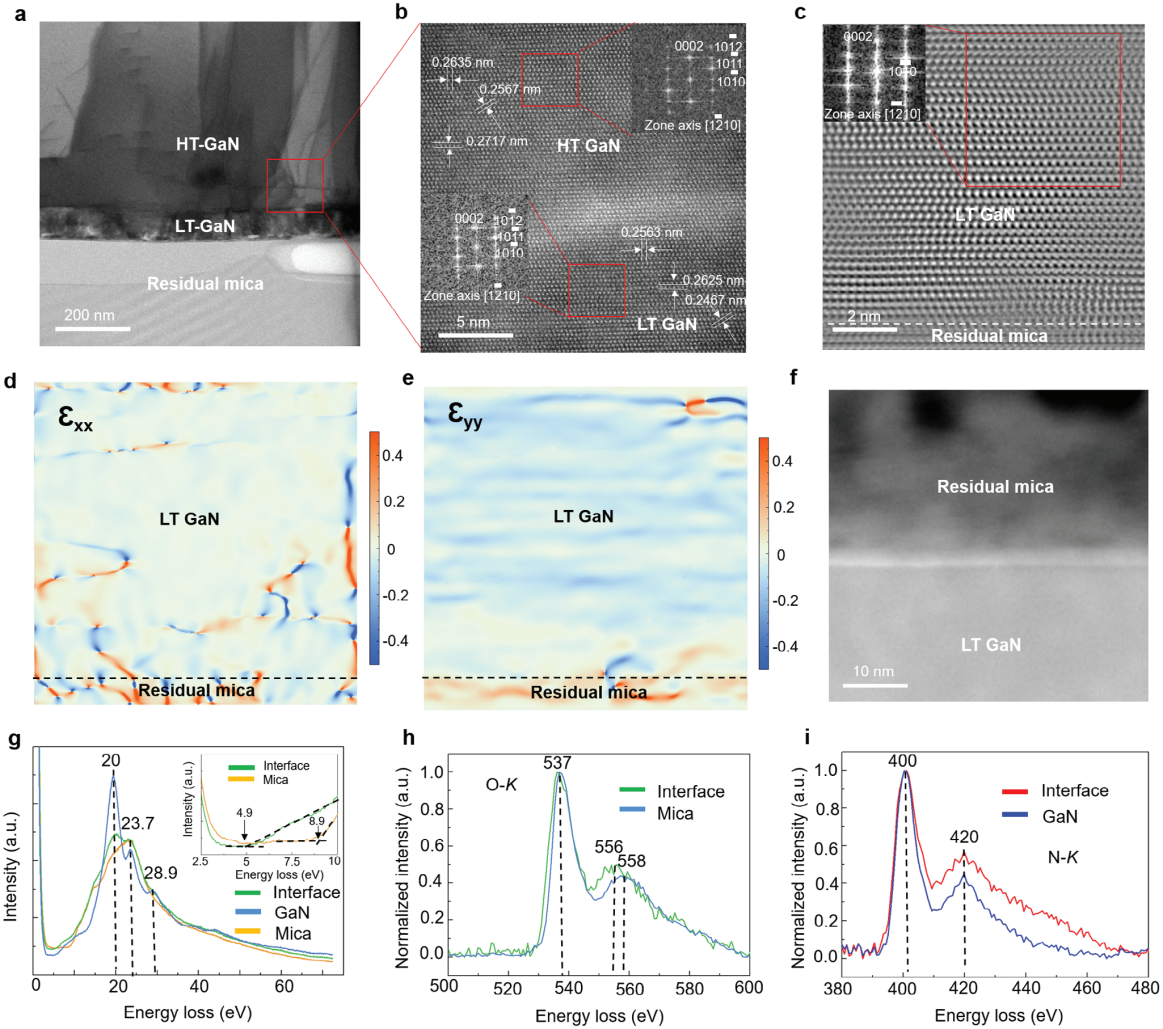
TEM images of self‐separated thick GaN film with residual mica. a) Low‐magnification TEM image of the back side of the self‐separated GaN with residual mica. b) High‐magnification TEM image of the interface between LT and HT GaN of the self‐separated GaN. c) STEM image of the interface at the back side of the self‐separated GaN and residual mica. d,e) *Ɛ*_xx_ and *Ɛ*_yy_ in‐plane strain mapping obtained from (c), respectively. f) Low‐loss electron energy loss spectroscopy (EELS) image at the interface between GaN and residual mica. g) Low‐loss EELS spectra extracted from (f). The inset shows the bandgap calculated using the linear fit method. The bandgap refers to the intersection point where the regression lines from the least‐square fitting cross the horizontal lines. h,i) O‐ and N‐*K* edge EELS spectra extracted from (f), respectively.

A further in‐depth investigation of the interface between residual mica and GaN was performed using high‐resolution transmission electron microscopy (HRTEM). As shown in the TEM image (**Figure** **3**a), the residual mica adhered exactly to the back side of the self‐separated thick GaN film. The adhered residual mica exhibits a satisfactorily uniform thickness, ranging from 157 nm to 1.5 µm (see Figure S9, Supporting Information), which is calculated to represent 157 to 1500 mica layers,^[^ ^41^ ^]^ respectively. Based on the diffraction patterns obtained from the HRTEM imaging (Figure 3b), we can confirm that HT GaN exhibits a single‐crystal structure and is grown along the c‐axis. To investigate the strain distribution at the interface between GaN and residual mica, a geometrical phase analysis (GPA) was performed. Figure 3c shows a scanning transmission electron microscopy (STEM) image of the GaN/mica interface. Figure 3d,e show the corresponding strain maps of *Ɛ*_xx_ and *Ɛ*_yy_ obtained from Figure 3c, respectively. The GPA results show no residual strain at the interface between GaN and residual mica, suggesting that the interface accommodates a significant portion of the residual strain during the vdWE epitaxy of GaN on mica. Although there is considerable lattice mismatch, calculated to be 46% (see Figure S10, Supporting Information), between mica and GaN, the interface could still be strain released.

We employed STEM in conjunction with electron energy loss spectroscopy (EELS) to investigate the chemistry and electronic structure of the pseudomorphic phase at the interface between residual mica and GaN. The resulting spatial distribution map, which can be seen in the low‐loss EELS (Figure 3f), clearly shows the presence of an interface phase, which can be electronically distinguished from both the GaN and the residual mica. Figure 3h,i show the EELS spectra of the O‐*K* and N‐*K* edges across the interface, respectively. Clearly, the atomic bonding of N at the interface is the same as that of bulk N (Figure 3i). This finding shows that N does not interact with residual mica other than through Ga–N interactions, which are necessary for the formation of the GaN layer. The O‐*K* edge characteristic peaks of both mica and interface are at 537 eV (Figure 3h), which corresponds to the O‐*K* ionization loss peak.^[^ ^42^ ^]^ The O‐*K* edge spectrum at the interface region exhibits a sub‐peak shift from 554 to 556 eV (Figure 3h). This is an interesting chemical feature of the interface where a noticeable amount of Ga reacts with the mica up to several nanometers in‐depth and forms gallium oxide. The band gap is found to be 4.9 eV with a gallium oxide thickness of 3 nm at the interface using the linear fit method (Figure 3g). Among the various phases of gallium oxide, β‐Ga_2_O_3_ has a bandgap of 4.9 eV,^[^ ^43^ ^]^ and a monoclinic structure with GaN has an in‐plane lattice misfit of 4.7%.^[^ ^44^ ^]^ According to recent reports, β‐Ga_2_O_3_ is a stable phase under ambient conditions, and the β‐Ga_2_O_3_ film can be epitaxially formed on a GaN substrate.^[^ ^45^, ^46^ ^]^ Based on these observations, we confirmed that the crystal structure of a pseudomorphically interface phase should be β‐Ga_2_O_3_. The mica layers at the interface were transformed into β‐Ga_2_O_3_, and there was self‐separation on the undamaged mica surface which was near β‐Ga_2_O_3_. The self‐separation process originates from the discrepancy in the thermal expansion coefficient and lattice constant between mica and GaN. The lattice constant mismatch between GaN and fluorophlogopite mica is up to 46%, with thermal expansion coefficients of 12 × 10^−6^ K^−1^ for mica and 5.59 × 10^−6^ K^−1^ for GaN. This mismatch induces significant strain between GaN and the mica substrate. Furthermore, the gradual formation of β‐Ga_2_O_3_ between the mica and GaN acts as a strain layer, adding to the stress between GaN and the mica substrate.^[^ ^47^ ^]^ The weak vdW bonding in the mica substrate also facilitates the self‐separation process.

### 2.4 Lift‐Off Process of Residual GaN from Mica Substrate

**Figure 4:**
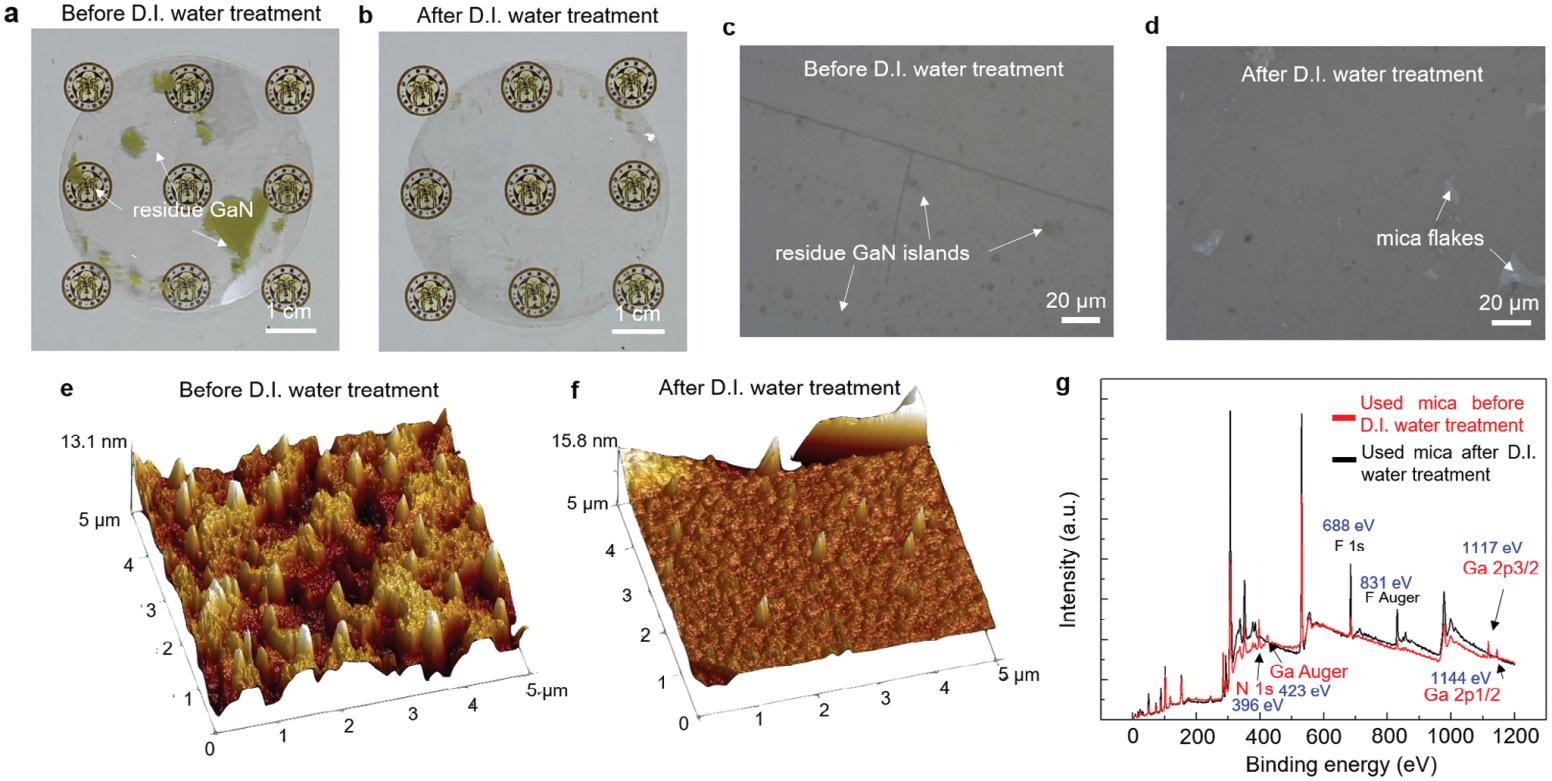
Lift‐off residue GaN from mica substrate on D.I. water treatment. a,b) Image of the mica substrate after GaN separation before and after D.I. water treatment at 90 °C for 4 h, respectively. c,d) Optical microscopy image of (a,b), respectively. e,f) 3D AFM image of (a,b), respectively. g) XPS spectra of the mica substrate before and after D.I. water treatment at 90 °C for 4 h.

We attempted to reuse the mica substrate to investigate the possibility of reducing the substrate cost. Although most of the two‐inch thick GaN film could self‐separate from the mica substrate intact, we selected a failed sample with a large area of residual GaN on the mica substrate after self‐separation for comparison (**Figure** **4**a). Owing to the hydrophilic characteristic of mica,^[^ ^48^ ^]^ most of the residual GaN on the mica substrate could be lifted off after immersing the substrate in D. I. water at 90 °C for 4 h, as shown in Figure 4b. Figure 4c,d show the optical microscopy images of the mica substrate before and after water treatment, respectively. The black dots in Figure 4c indicate several GaN residue islands, where the black GaN stripes originate from the nucleation on the 2D material stripes. After water treatment on the mica substrate, the residual GaN islands decreased in number and size, and the mica flakes were seldom lifted from the surface, as shown in Figure 4d. To further investigate the morphology of the mica surface before and after the water treatment, we captured the atomic force microscopy (AFM) images of the mica surface (Figure 4a,b), as shown in Figure 4e,f, respectively. From the root‐mean‐square (RMS) value of the AFM images shown in Figure 4e,f, the RMS value of the AFM image before water treatment is found to be higher than that after the treatment, indicating that the water treatment effectively lifts off the residual GaN from the mica substrate.

Figure 4g shows the survey‐scan XPS spectrum of the D.I. water‐treated mica substrate. All the element compositions of mica exhibit XPS signals, including O 1s (E_b_ ≈ 532 eV), F 1s (E_b_ ≈ 688 eV), Mg 2p (E_b_ ≈ 49 eV), Mg 2s (E_b_ ≈ 88 eV), Si 2s (E_b_ ≈ 152 eV), Al 2s (E_b_ ≈ 117 eV), Si 2p (E_b_ ≈ 101 eV), Al 2p (E_b_ ≈ 71 eV), and O 2s. An X‐ray‐induced O 1s energy‐loss peak can also be observed. A C 1s peak (E_b_ ≈ 286 eV) caused by surface contamination can be clearly seen because the mica surface easily attracts carbonaceous contaminants from the ambient environment.^[^ ^49^ ^]^ The residual GaN exhibits peaks at Ga 3d (E_b_ ≈ 19 eV), Ga 2p_1/2_ (E_b_ ≈ 1144 eV), Ga 2p_3/2_ (E_b_ ≈ 1117 eV), and N 1s (E_b_ ≈ 396 eV) in the XPS spectra both before and after the D.I. water treatment of the mica substrate, revealing that some of the residual GaN islands remained on the mica surface after the treatment. Furthermore, the intensity of the Ga and N peaks after the D.I. water treatment was lower than that before the treatment, indicating that some of the residual GaN islands were lifted off after the treatment. The F 1s (Eb ≈ 688 eV) and F Auger (E_b_ ≈ 831 eV) both remained on the XPS spectrum; this suggests that LT GaN can efficiently protect the mica surface from ammonia damage and also proves that there is no chemical damage to the mica surface during D.I. water immersion.

### 2.5 Mica Substrate Recycling for GaN Reproduction

**Figure 5:**
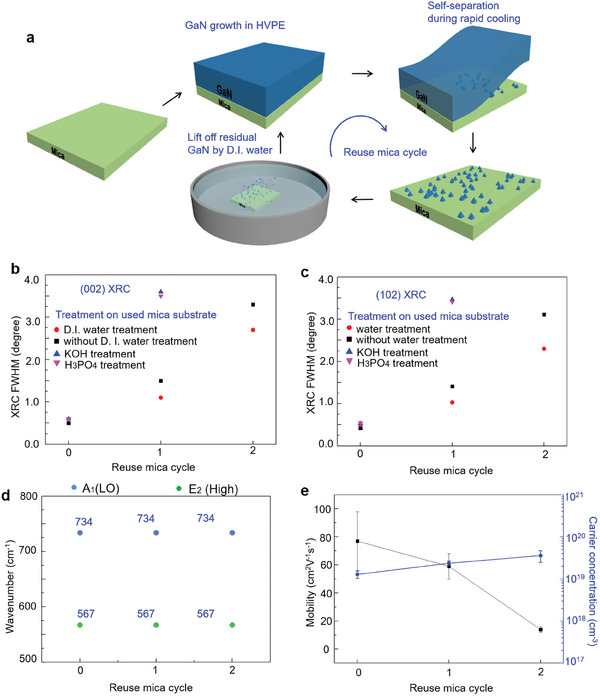
Reproduction process of growing self‐separated thick GaN films on used mica substrate. a) Schematic of the fabrication of self‐separated thick GaN film on a reused mica substrate. b,c) (002) and (102) XRC of the self‐separated thick GaN film grown on various treated pristine substrates and used mica substrates, respectively. d) Raman spectrum of self‐separated thick GaN film grown on pristine substrate and mica substrates subjected to various reuse cycles. e) Hall mobility and carrier concentration of the self‐separated thick GaN film grown on pristine substrate and mica substrate used subjected to various reuse cycles measured at room temperature.

We examined the quality and residual strain of the separated thick GaN film on mica substrates subjected to various reuse cycles to establish the maximum number of times the same mica substrate can be reused. **Figure** **5**a shows a schematic of the procedure of the growth cycles of the self‐separated thick GaN film using reused mica substrates. As shown in Figure 5b,c, the FWHM of the (002) XRC of the as‐grown self‐separated GaN enhances with the increase in the reuse cycle of the mica substrate, and the FWHM of the (102) XRC also presents the same trend as that of the (002) XRC. Apparently, the quality of GaN grown on D.I. water‐treated used mica is better than that of GaN grown on the used mica substrate untreated by water. This result is explained by the increase in the number of residual GaN islands contributing to more noneliminated threading dislocations during GaN growth, causing a substantial degradation in the GaN quality. Although we attempted to use H_3_PO_4_ and KOH to etch the residual GaN on the mica substrate and then reuse the mica substrate, the FWHM of the regrown GaN increased substantially because of the surface damage to the mica substrate (Figure S11, Supporting Information) induced during the aforementioned procedures. We investigated the residual strain in the regrown self‐separated GaN using Raman spectroscopy, as shown in Figure 5d. Noticeably, both E_2_ (high) and A_1_ (LO) peaks remain at 567 and 734 cm^−1^, respectively, in all the GaN samples, indicating that no residual strain occurred in the regrown GaN separated from the mica substrate. We examine the XPS spectra of the self‐separated GaN thick films. The surface state of the regrown GaN is similar to that of the GaN film grown on pristine mica (Figure S12, Supporting Information). We also capture TEM images and GPA mappings of the interface between regrown GaN and reused mica substrate (Figure S13, Supporting Information). The fluorophlogopite mica substrate used in our experiment is single crystalline, as evidenced by the diffraction patterns in the TEM image. However, the mica surface can be damaged after repeated usage due to prolonged exposure to NH_3_ and HCl during the nitridation and GaN growth steps. Additionally, fluorophlogopite mica is sensitive to the accelerated electron beam in the TEM system, which may cause beam damage to the mica surface, leading to its transformation into an amorphous structure.

Hall measurements were conducted to determine the electrical properties of the layers on the self‐separated thick GaN film. Figure 5e shows the Hall mobility and carrier concentration of the separated thick GaN film, grown to a similar thickness of 483 mm. The self‐separated thick GaN films exhibited a lower mobility and higher carrier concentration, suggesting that these thick films contain impurities and that these unintentionally doped GaN crystals exhibit n‐type conductivity (with mainly O as donors, from the PL spectrum). In a high‐temperature growth environment, the mica substrate cleavages and provides large amounts of O impurities for GaN. The mobility reduces when the reuse cycle increases and this trend is consistent with the quality of the self‐separated thick GaN film. Small differences are evident in the mobility and carrier concentration of the samples grown in the process, and these are due to the differences in the growth rate. The reason for this is the reduction in dislocation scattering because the dislocation density decreases with the thickness. The carrier concentration of the self‐separated thick GaN film is 1.3 × 10^19^ cm^−3^, which is 2 orders higher than that of the thick GaN film grown on sapphire using the HVPE method. The best Hall mobility recorded is 98 cm^2^ Vs^−1^ at room temperature, which is lower than that of the thick GaN film grown on sapphire through the HVPE method.^[^ ^50^ ^]^

### 2.6 Demonstration of UV‐LED on Self‐Separated Thick GaN Film

**Figure 6:**
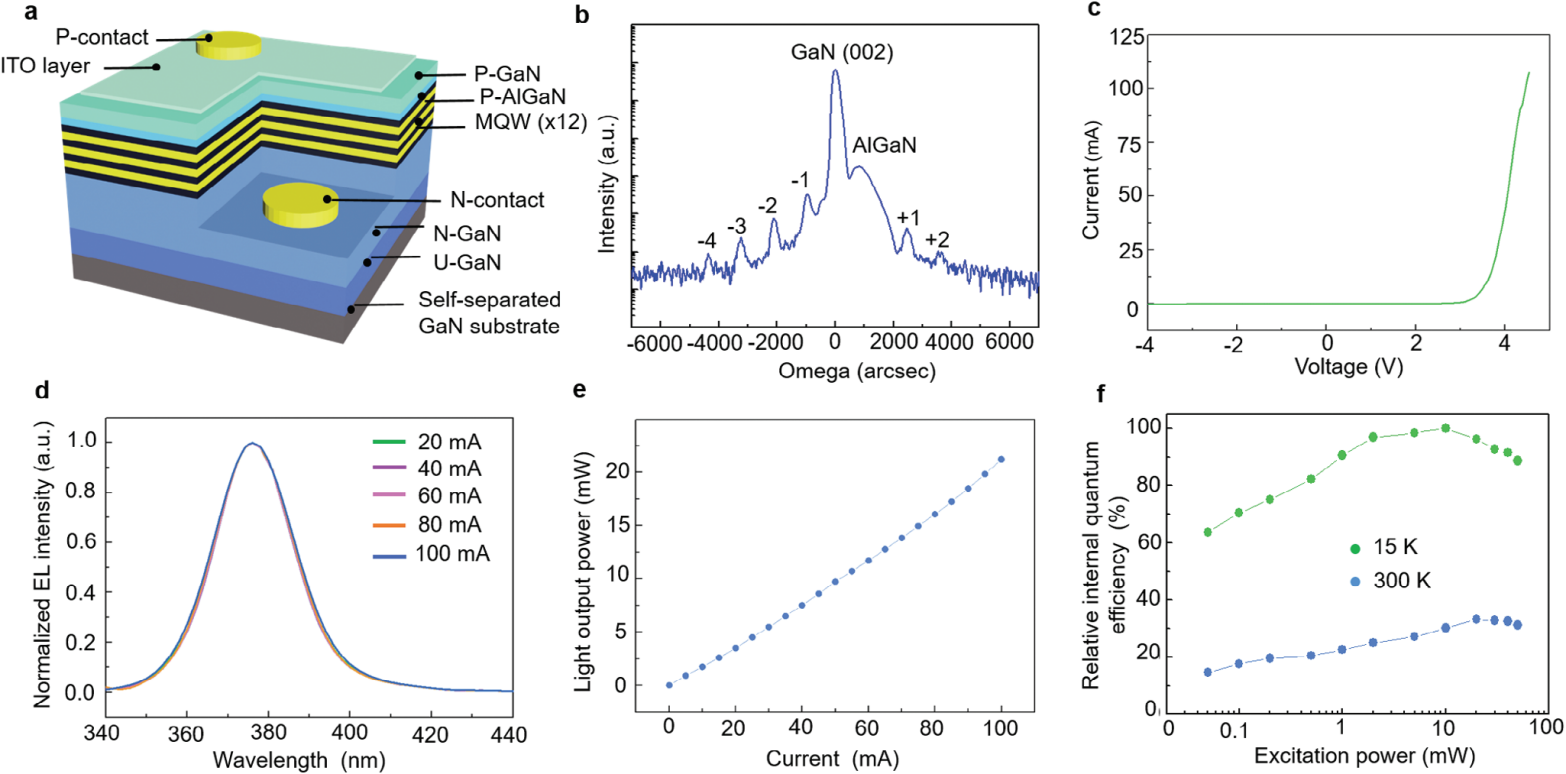
Structure and characterizations of UV‐LEDs grown on self‐separated GaN substrates. a) Schematic of UV‐LED on a self‐separated GaN substrate. b) X‐ray omega‐scan of UV‐LED grown on a self‐separated GaN substrate. c) *I–V* characteristic of the UV‐LED device. d) Normalized EL intensity versus wavelength. e) LOP plot of UV‐LED as a function of the injection current. f) Relative IQE as a function of the injected current.

Such free‐standing GaN films separated from a mica substrate can enable LED fabrication. **Figure** **6**a shows the schematic of the fabrication process of the GaN UV‐LED structure. The XRD omega‐scan in Figure 6b shows intense satellite peaks in the UV‐LED heterostructure on the self‐separated GaN substrate, indicating the formation of high‐quality MQWs. The TEM image also demonstrates the UV‐LED device on self‐separated GaN, showcasing high‐quality MQWs (Figure S14, Supporting Information). Furthermore, the current‐voltage curve of the UV‐LED shows a turn‐on voltage of 3.25 V and a low leakage current of 0.04 mA at −4 V (Figure 6c). The electroluminescence (EL) characteristics of the UV‐LEDs were further evaluated, as shown in Figure 6d. The UV‐LED showed no shift in peak position with increasing current to 100 mA. The light output power (LOP) of the UV‐LED on the self‐separated thick GaN film exhibited a linear relationship with the input current, with a slope efficiency of ≈0.21 mW mA^−1^, indicating that the EL emission was generated from radiative recombination and carrier injection at the MQW layers (Figure 6e). The relative external quantum efficiency (EQE) as a function of the injection current is shown in Figure S15, Supporting Information. Under a strain‐free condition, the relative EQE first increases with increasing injection current and then significantly drops with the increase in the current after reaching its maximum value at a current of 25 mA. To gain further insights into the mechanism of luminescence enhancement of the UV‐LED, a temperature‐dependent internal quantum efficiency (IQE) measurement was performed as shown in Figure 6f. When the power density was low, the IQE revealed a distinct enhancing trend with increasing power density. This was attributed to the consequence of point defects screening the nonradiative recombination centers. With increasing power density, the injected carriers would gradually compensate for these point defects, thereby raising the IQE value. In contrast, as the power density was high, the IQE revealed an evidently reducing trend, which was described as the undesired “efficiency droop effect.” These results demonstrate that highly efficient UV‐LEDs can be fabricated on a self‐separated GaN substrate. As shown in **Table** **1**, our experiment shows an obvious demonstration for producing wafer‐scale self‐separated GaN and the reusability of the original substrate compared with previous research, expanding the substrate choice in III‐V self‐separation technology. Meanwhile, our proposed self‐separation of GaN from mica possesses a remarkable 90 percent success rate of self‐separation.

**Table 1:** Comparison of free‐standing GaN fabrication reported in previous research.

Structure	Lift‐off method	(002) XRC (arcsec)	(102) XRC (arcsec)	Wafer scale	LED demonstration	Substrate reusability	Refs.
GaN (HVPE)/mica	Self‐separation	1800	1584	2‐inch	UV‐LED	2 times	This work
GaN (MOCVD)/hBN/GaN	Tape‐assisted	N/A	N/A	2‐inch	N/A	2 times	[14]
GaN (MOCVD)/sapphire	Laser lift‐off	N/A	N/A	N/A	Yes	N/A	[15]
GaN (HVPE)/SiC	Self‐separation	261	272	N/A	N/A	N/A	[51]
GaN (HVPE)/GaN(MOCVD)/sapphire	Self‐separation	49	65	2‐inch	N/A	N/A	[52]
GaN (HVPE)/GaN(MOCVD)/sapphire	Chemical etching	226	376	2‐inch	N/A	N/A	[53]
GaN (MOCVD)/sapphire	Laser lift‐off	N/A	N/A	N/A	Yes	N/A	[54]
GaN (MOCVD)/ZnO/sapphire	Chemical‐lift off	N/A	N/A	N/A	N/A	N/A	[55]
GaN (HVPE)/AlN/GaAs	Self‐separation	695	350	2‐inch	N/A	N/A	[56]

## 3 Conclusion

In summary, we investigated the ambient effect on the surface of a mica substrate used to fabricate high‐quality GaN under optimized growth conditions. We fabricated 2‐inch thick GaN films on a fluorophlogopite mica substrate and obtained thick GaN films that could self‐separate from the substrate during rapid cooling. The 2‐inch single‐crystal GaN films could be grown up to 483 µm thick with a rapid growth rate of ≈97 µm h^−1^. After the growth of self‐separated GaN, the residual GaN on the separated mica substrate could be efficiently removed by immersing it in D.I. water. Further, we successfully demonstrated the reusability of the mica substrate by growing self‐separated thick GaN films repeatedly on the used mica substrate. These as‐fabricated thick GaN films showed a completely strain‐free characteristic. A series of LED characterizations showed that highly efficient UV‐LEDs can be grown on free‐standing GaN self‐separated from a mica substrate. This work reveals the effective integration between large‐area grown GaN and mica and motivates further research on the possibility of expanding substrate choice in GaN self‐separation techniques.

## 4 Experimental Section

### Epitaxial Growth of Self‐Separated Thick GaN Film on Mica Substrate

Figure 1a presents a schematic representation of the growth process for self‐separated thick GaN films using the HVPE method. Two‐inch fluorophlogopite mica was used as the substrate for the growth, and the substrate thickness was 32 ± 5 µm. A two‐step growth process was introduced to protect the mica surface from thermal damage and to reduce the lattice mismatch. The mica substrate was first heated to 600 °C, accompanied by nitridation for 10 min. Simultaneously, HCl gas was made to react with liquid Ga at 850 °C to produce GaCl gas, which was subsequently carried to the reactor accompanied by H_2_/N_2_ carrier gas. Subsequently, HCl and NH_3_ were induced to synthesize LT GaN for 10 min at 600 °C with a V/III ratio of 14.89. The samples were then heated to 950 °C under ambient NH_3_ conditions to prevent LT GaN from dissociating at high temperatures. The sample was nitridated for 10 min at 950 °C, followed by the synthesis of HT GaN for 5 h. HCl gas (flow rate: 67 sccm) and NH_3_ gas (flow rate: 2.5 slm) were used as precursors in the HT GaN fabrication. A gas mixture (50% of hydrogen in nitrogen) was used as the carrier gas during all the HVPE processes. After HT GaN growth, the thick GaN films were self‐separated during the rapid cooling process under N_2_ ambient conditions. All the experiments were conducted at a total pressure of 700 Torr. After the GaN self‐separated from the mica substrate, the used mica substrate was immersed in D.I. water for 4 h at 90 °C and this process lifted off most of the residual GaN from the mica substrate. For the regrowth of GaN on the used mica substrate, the immersed mica substrates were then dried and once again subjected to the HVPE system. The growth procedure used for the LT/HT GaN was the same as that used previously.

### Epitaxial Growth of LED Structure using the MOCVD Method

The thickness of the polished self‐separated GaN used for the LED substrate was 400 µm. It is important to note that the polishing treatment may reduce the thickness of the original GaN thick film. During the growth process of nitride films using the MOCVD method, trimethylaluminum (TMAl), trimethylgallium (TMGa), trimethylindium (TMIn), and NH_3_ were adopted as Al, Ga, In, and N precursors, respectively. Silane (SiH_4_) and magnesocene (Cp_2_Mg) were adopted as n‐ and p‐doped sources, respectively. Throughout the growth process, H_2_ acted as a carrier gas. The LED structure comprised a 2 µm µ‐GaN layer, a 2.5 µm Si‐doped GaN layer, 12 periods of InGaN/AlInGaN multiple quantum wells (MQWs), a 15 nm Mg‐doped AlGaN electron‐blocking layer, and a 100 nm Mg‐doped GaN layer. Each period of the MQW comprised a 12.5 nm AlInGaN barrier layer and a 2.5 nm InGaN well.

### Process of LED Device Fabrication

An indium tin oxide layer (300 nm) was deposited on the p‐GaN layer by electron beam evaporation. Photolithography and inductively coupled plasma etching were utilized to expose the n‐GaN layer. Ti/Al/Ti/Au and Ni/Au were adopted as the *p*‐ and *n*‐type contact electrodes by electron beam evaporation, respectively. The size of our LED was 300 × 300 µm^2^.

### Material Characterization

SEM images were captured utilizing a Hitachi SU‐8010 system operated at 15 kV to study the surface morphology. EBSD characterization was performed using an FEI Quanta SEM equipped with an EBSD detector (Oxford Instruments). The maps were analyzed using the Oxford Aztec and Tango software programs. The TEM lamella for cross‐sectional observation was prepared in the TESCAN GAIA3 dual‐beam focus ion beam‐SEM system. HRTEM images and EELS spectra were performed using a Cs‐corrected JEOL ARM200F TEM operated at 80 and 200 kV, respectively. The XPS measurements were conducted on a multi‐technique 100 mm hemispherical electron analyzer (PHI Quantera II), utilizing Al Kα radiation as the excitation source. The sample morphology was performed using AFM in the contact mode (MMSPM NanoScope IV) with Si_3_N_4_ tips.

## Conflict of Interest

The authors declare no conflict of interest.

## Supporting information

Supporting Information

## Data Availability

The data that support the findings of this study are available in the supplementary material of this article.
