# Supplementary material for: Direct Growth of Wafer‐Scale Self‐Separated GaN on Reusable 2D Material Substrates
Source: Adv Sci (Weinh). 2024 Sep 3;11(41):2406126. doi: 10.1002/advs.202406126 (PMC11538651; doi:10.1002/advs.202406126)
Supplement: Supplementary file 1 — Supporting Information [file ADVS-11-2406126-s001.docx]

**Supporting Information**

Direct growth of wafer-scale self-separated GaN on reusable two-dimensional material substrate

Chang-Hsun Huang^1^, Chia-Yi Wu^1^, and Yi-Chia Chou^1,*^

^1^Department of Material Science and Engineering, National Taiwan University, Taipei 10617, Taiwan

*Corresponding author E-mail: [ycchou@ntu.edu.tw](mailto:ycchou@ntu.edu.tw)


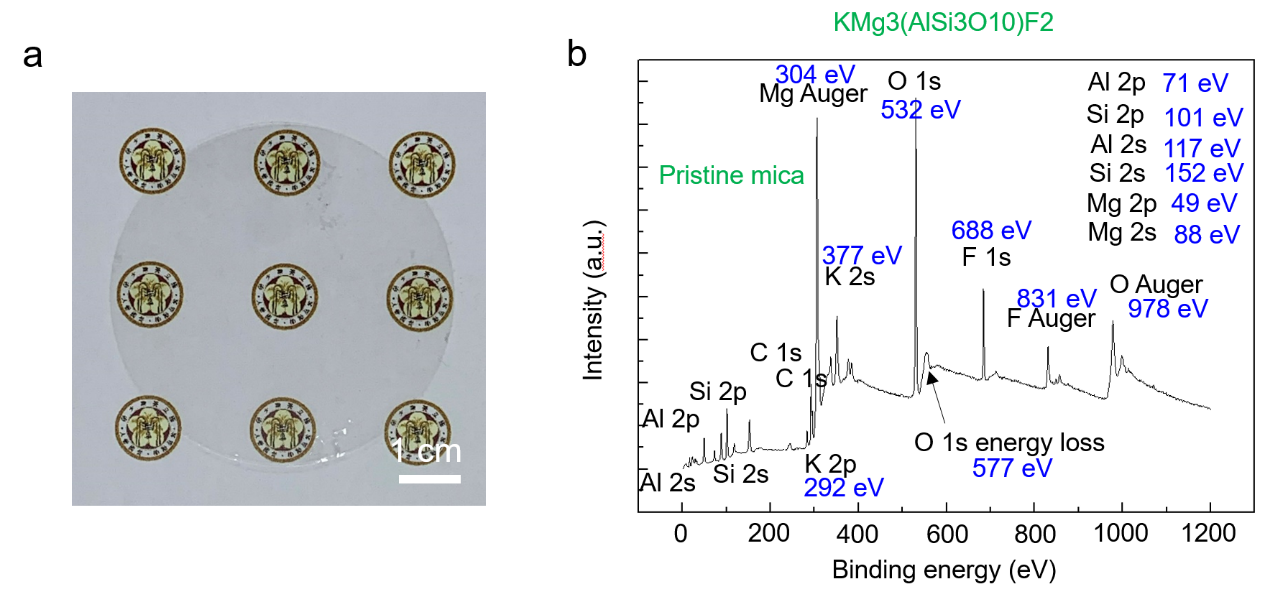


**Figure S1. OM image and XPS spectrum of pristine mica.** (a) Image of a 2-inch pristine mica substrate. (b) XPS spectrum of a pristine mica surface.


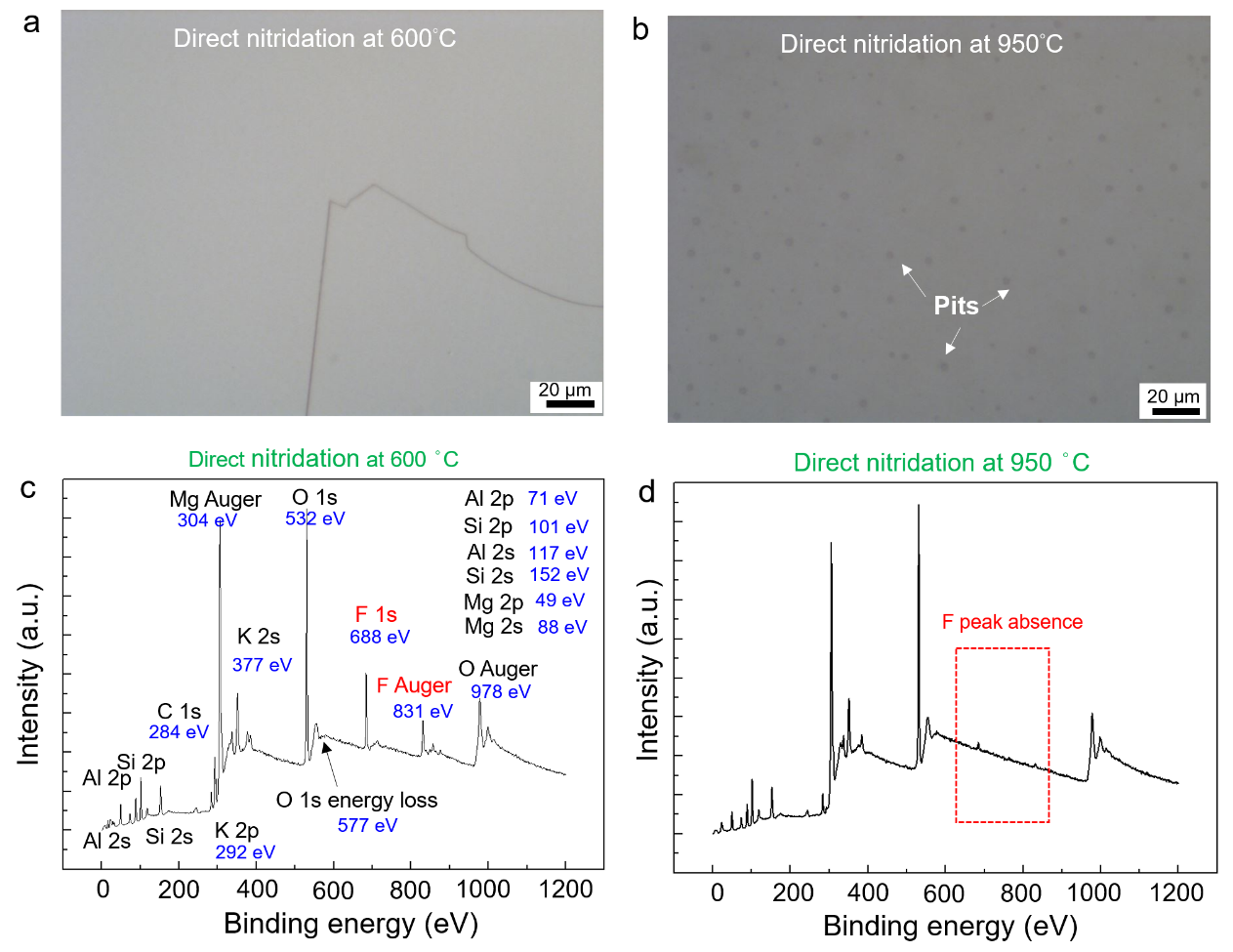


**Figure S2. The influence of nitridation process on the surface of mica substrate.** (a, b) Optical microscopy images of the surface of mica substrates after 10 min nitridation at 600 and 950 ºC, respectively. Several pits are observed at mica substrate after 10 min of nitridation at 950 ºC. (c, d) XPS spectra of mica substrates after 10 min nitridation at 600 and 950 ºC, respectively.


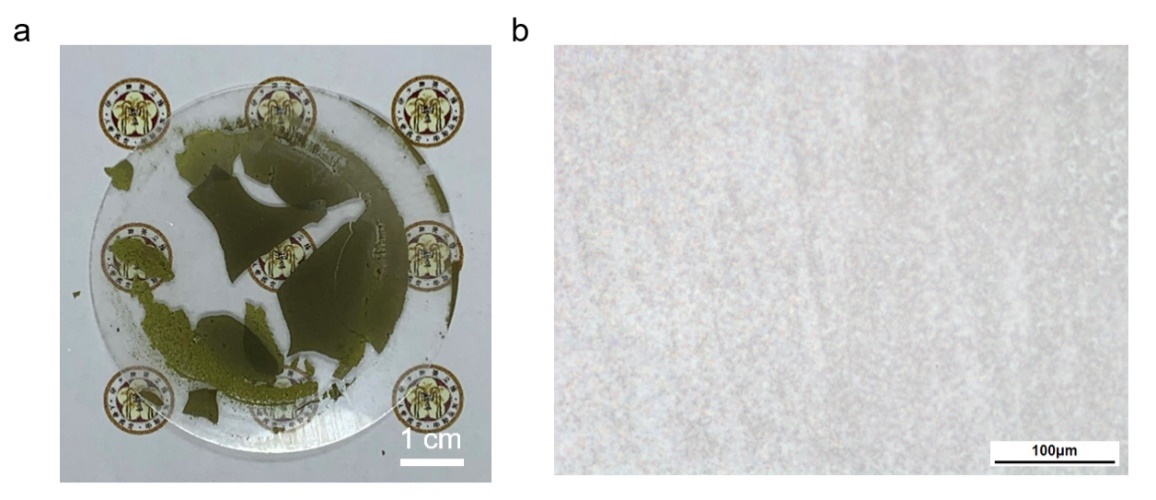


**Figure S3.** **The morphology of mica substrate after GaN growth at 1000 ºC.** (a) Image of the mica substrate after GaN growth reveals that the self-separated GaN film barely remains intact after being directly grown on the mica substrate. (b) OM image of the surface of the mica substrate after GaN growth.

**
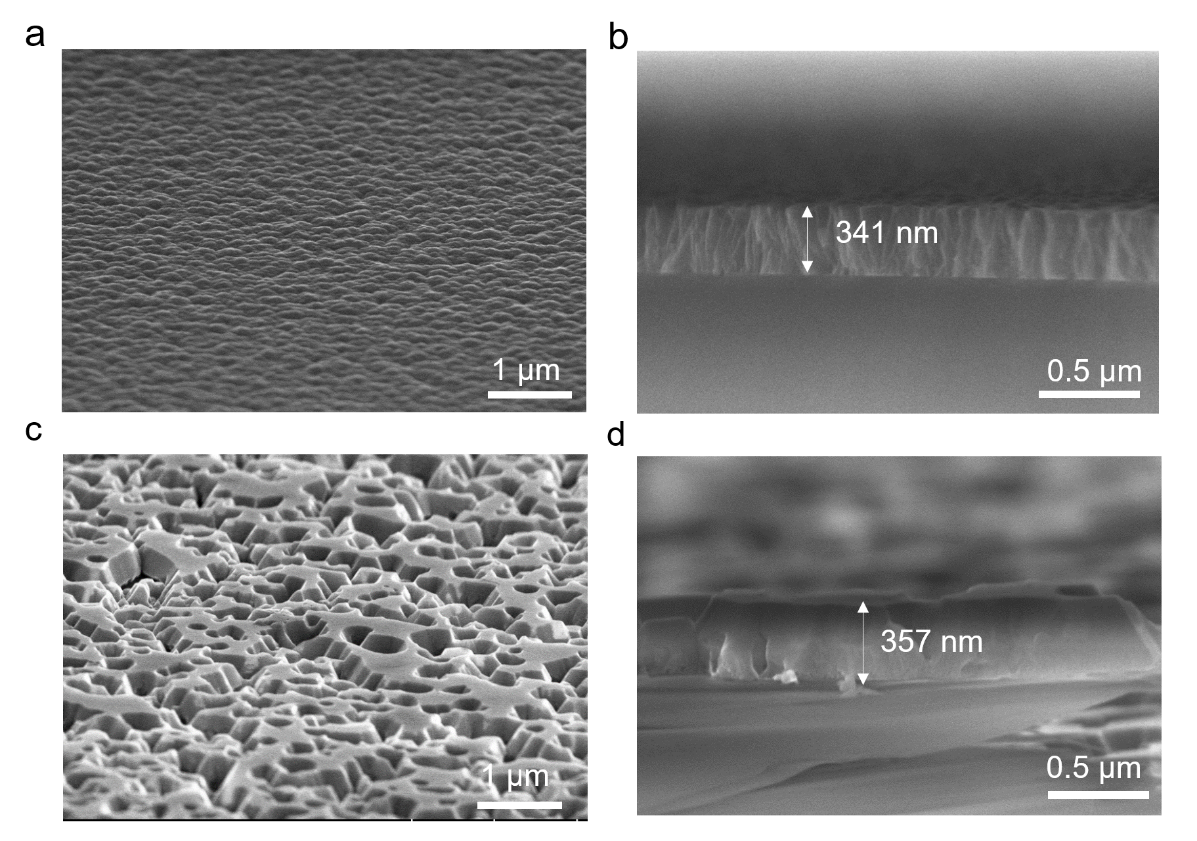
**

**Figure S4. The morphology of LT-GaN grown at 600 °C for 10 min.** (a, b) Plane-view and cross-section SEM images of as-grown LT-GaN on mica substrate, respectively. (c, d) Plane-view and cross-section SEM images of LT-GaN on mica substrate after 30 min annealing at 950 °C, respectively.

**
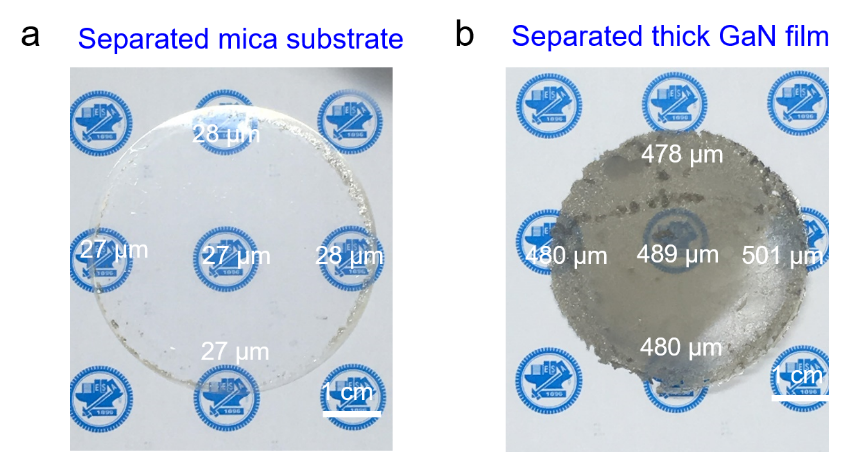
**

**Figure S5. The thickness measurements for both the self-separated thick GaN film and the used mica substrate.** (a) The used mica substrate has an average thickness of 27.4 μm. (b) The average thickness of the self-separated GaN film is measured at 483 μm.


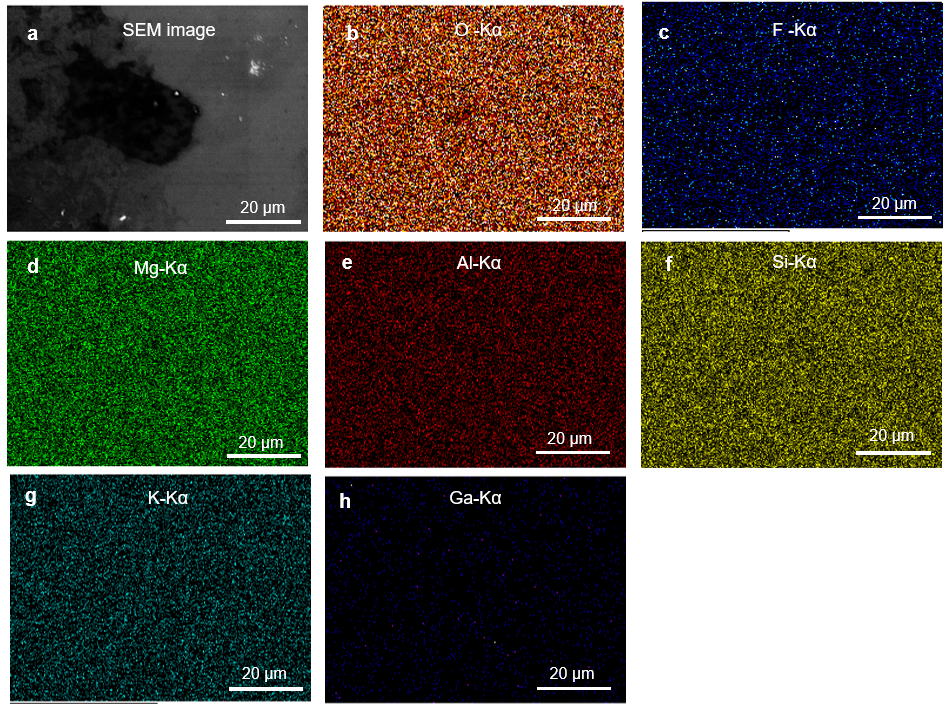


**Figure S6.** **EDS mapping of the back side of self-separated GaN thick film.** (a) SEM image of the back side of self-separated GaN thick film. (b-h) EDS mapping for O, F, Mg, Al, Si, K, and Ga elements of (a), respectively.


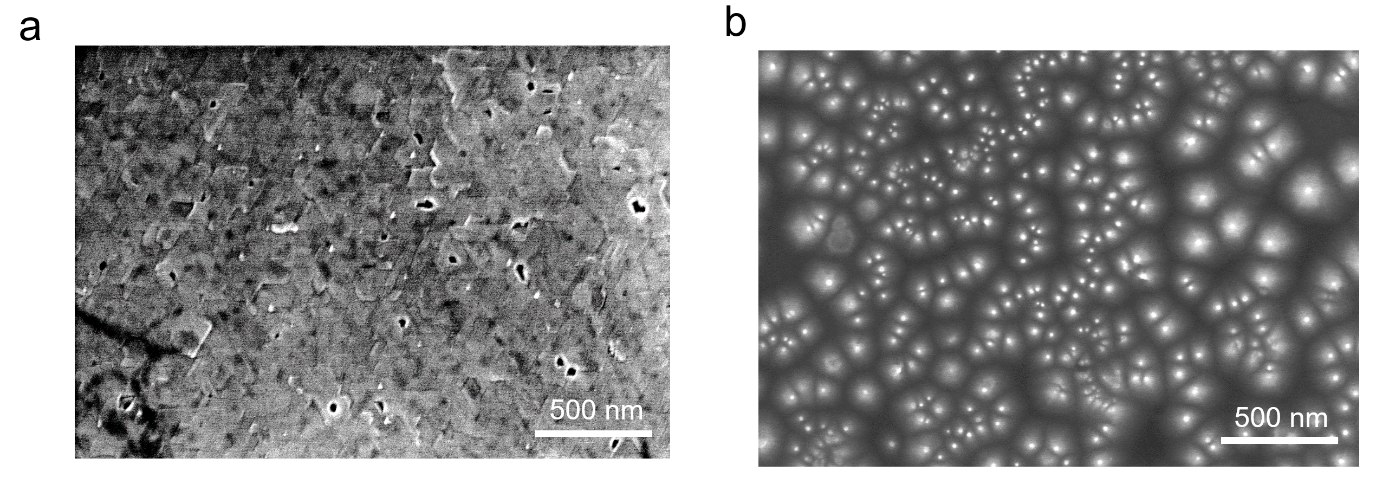


**Figure S7. The morphologies of the front and back surface after phosphoric acid etching.** (a, b) The SEM images of front side and back side of self-separated GaN thick film after phosphoric acid etching at 160 °C for 5 min, respectively.

**
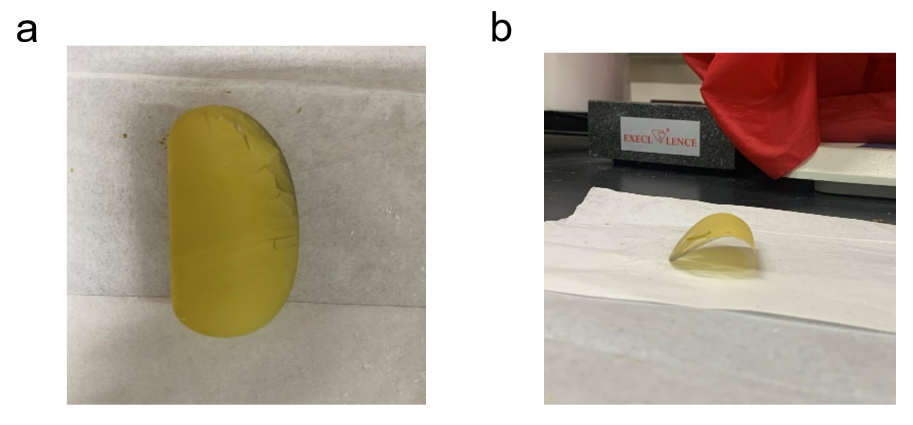
**

**Figure S8. As-grown GaN film grown on mica substrate without rapid cooling process.** (a, b) Plane view and cross-section view of a 2-inch GaN film grown on mica substrate without rapid cooling process, respectively.


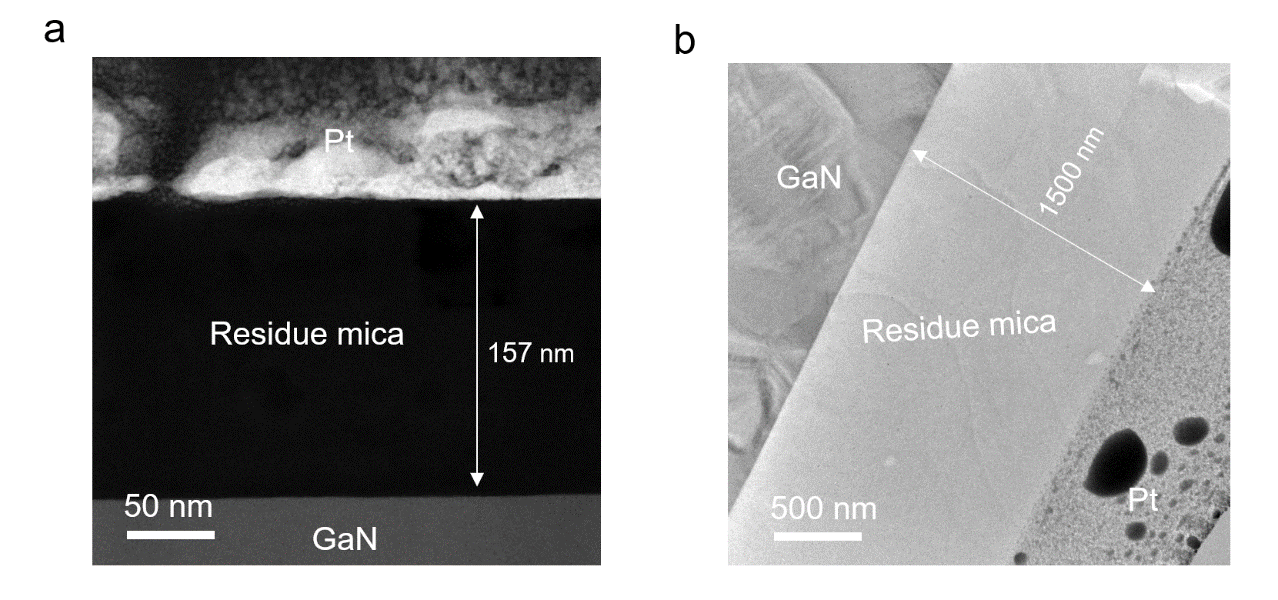


**Figure S9. The thickness of residue mica adhered to the backside of self-separated GaN thick film.** (a, b) Low-magnification TEM images of the attached residual mica exhibit satisfactorily uniform thickness of approximately 157 nm and 1.5 μm, respectively.

**
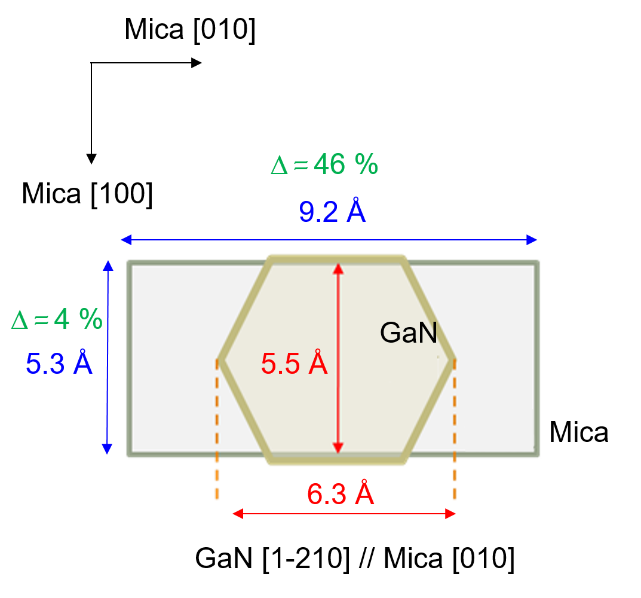
**

**Figure S10. The calculation of lattice mismatch between mica and GaN.** The lattice mismatch between mica and GaN is calculated to be 46 %.

**
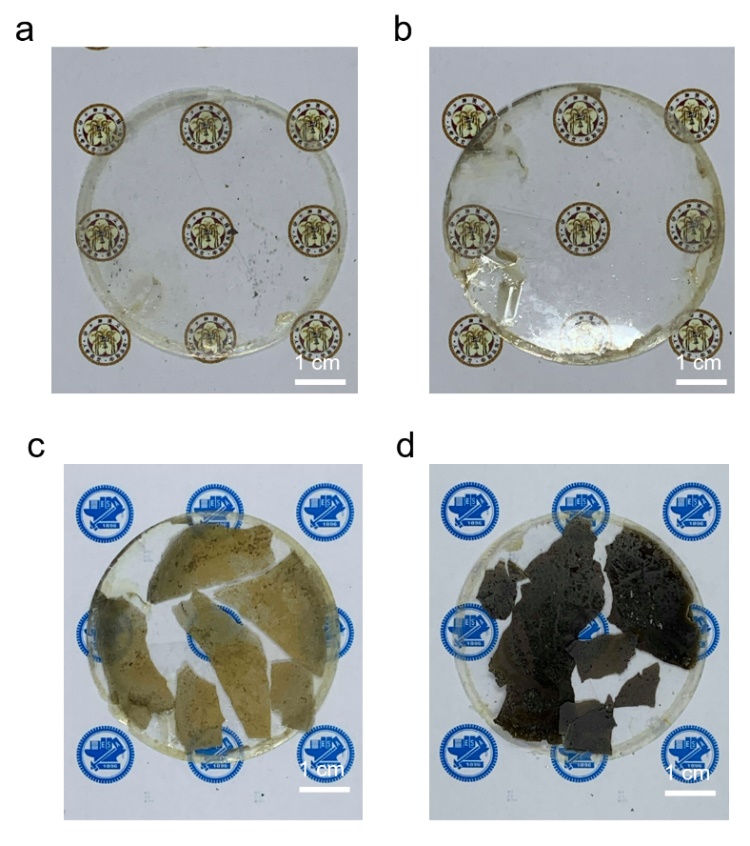
**

**Figure S11. The morphology of used mica substrate after H_3_PO_4_ and KOH treatments.** (a, b) Image of the used mica substrate after H_3_PO_4_ and KOH treatment, respectively. (c, d) Self-separated GaN film grown on H_3_PO_4_ and KOH-treated mica substrate, respectively.


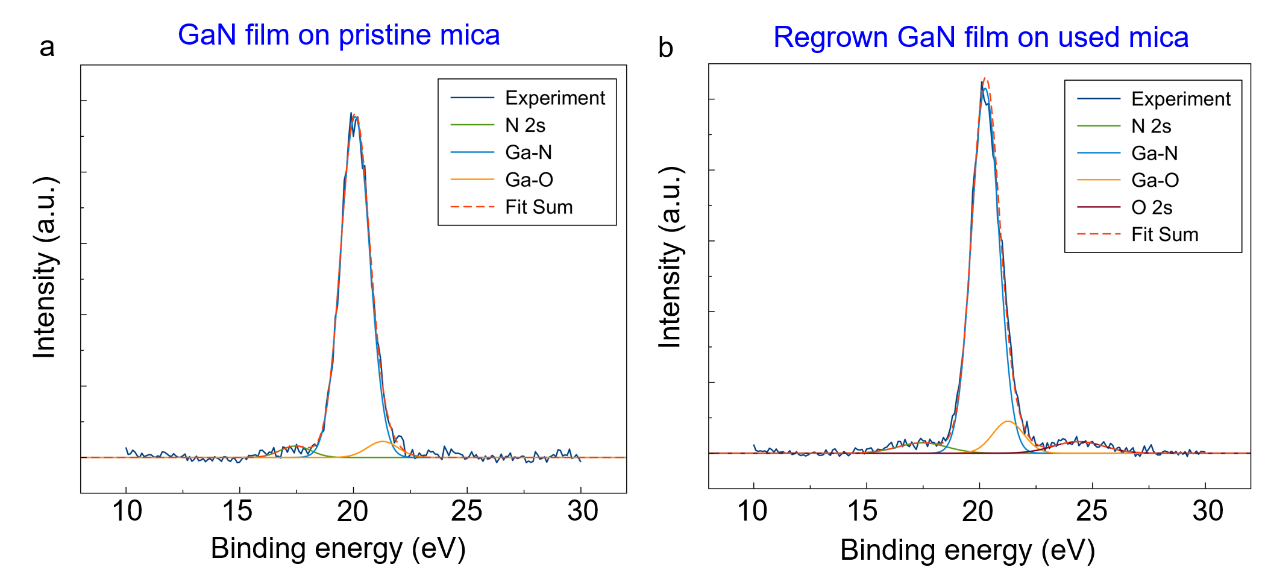


**Figure S12. XPS spectra of the self-separated GaN thick films.** (a, b) Ga 3d of the GaN thick film grown on pristine mica and the regrown GaN thick film, respectively. The Ga-3d core-level line contains four subpeaks. The main peak originates from Ga–N bonds, while the other two correspond to N 2s and Ga–O bonds, with a slight subpeak resulting from the overlap of the O 2s state.


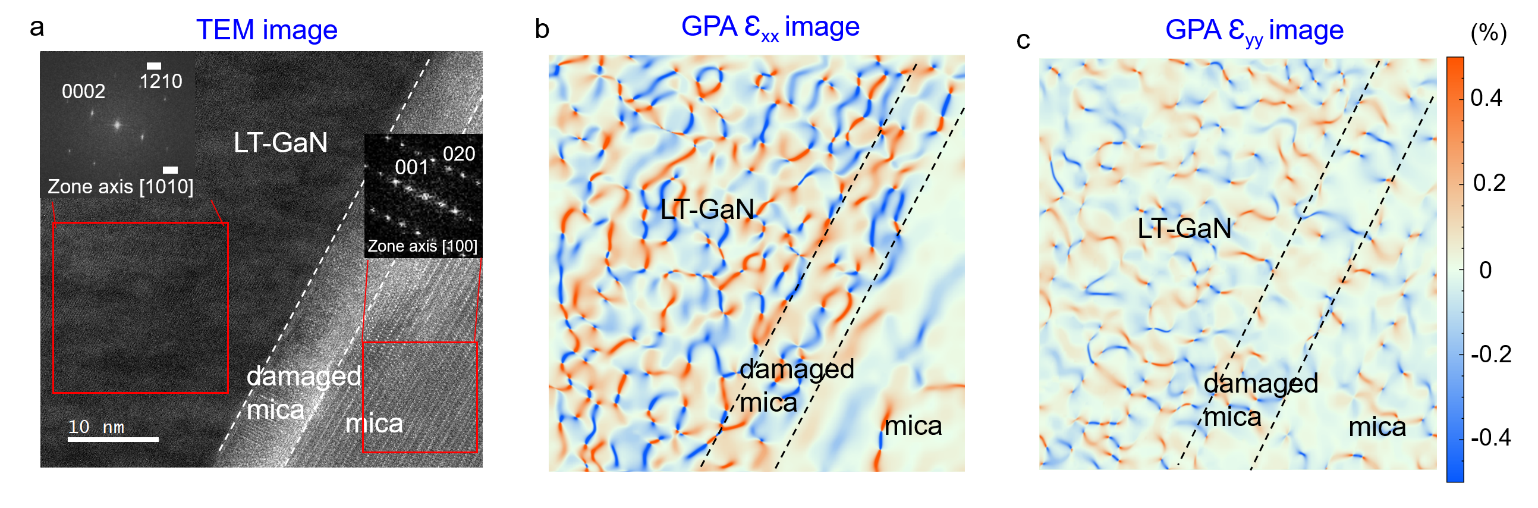


**Figure S13.** **TEM image and GPA mappings of the interface between LT-GaN and the reused mica substrate.** (a) TEM image of the interface between LT-GaN and the reused mica substrate. (b, c) *Ɛ*_xx_ and *Ɛ*_yy_ in-plane strain mapping obtained from (a), respectively.


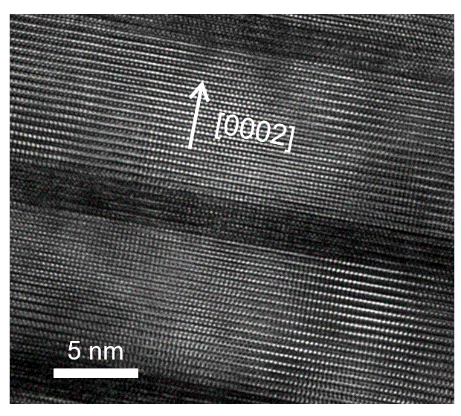


**Figure S14. TEM image of the MQWs of the UV-LED on a self-separated GaN substrate.** The TEM image shows the MQWs with high uniformity.


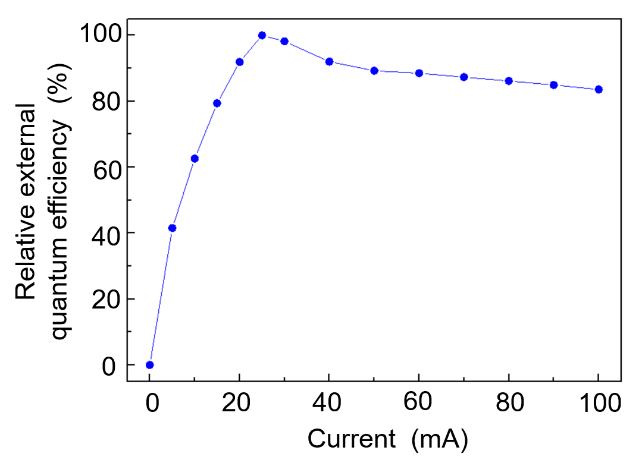


**Figure S15. Relative internal quantum efficiency as a function of the injected current.** The relative external quantum efficiency initially rises with an increase in injection current, reaching its peak at a current of 25 mA, followed by a decline as the current continues to increase beyond this point.
